# Prognostic and clinicopathological value of soluble programmed cell death ligand-1 (sPD-L1) in patients with peripheral T-cell lymphoma: a meta-analysis

**DOI:** 10.1080/07853890.2025.2458236

**Published:** 2025-02-10

**Authors:** Ying Wu, Yan Zhang

**Affiliations:** Department of Hematology, Huzhou Central Hospital, Affiliated Central Hospital of Huzhou University, Huzhou, Zhejiang, China

**Keywords:** Soluble PD-L1, peripheral T-cell lymphoma, meta-analysis, prognosis, biomarker

## Abstract

**Background:**

Previous studies have explored whether soluble programmed cell death ligand-1 (sPD-L1) can be used to predict the prognosis of patients with peripheral T-cell lymphoma (PTCL); however, no consistent results have been obtained. Consequently, we conducted the present meta-analysis to identify the precise significance of sPD-L1 in predicting the prognosis of PTCL.

**Methods:**

We searched PubMed, Embase, Web of Science, and the Cochrane Library until July 31, 2024. The value of sPD-L1 in predicting PTCL prognosis was examined by combining the hazard ratios (HRs) with 95% confidence intervals (CIs).

**Results:**

Seven articles involving 445 patients were included in this study. Based on our pooled findings, increased sPD-L1 was associated with dismal overall survival (OS) (HR = 4.22, 95%CI = 1.89–9.43, *p* < 0.001) and worse progression-free survival (PFS) (HR = 2.57, 95%CI = 1.35–4.90, *p* = 0.004) in PTCL. Furthermore, higher sPD-L1 levels were correlated with male sex (OR = 1.80, 95%CI = 1.06–3.03, *p* = 0.029), International Prognostic Index (IPI) score ≥2 (OR = 4.32, 95%CI = 2.10–8.89, *p* < 0.001), elevated lactate dehydrogenase (LDH) level (OR = 5.15, 95%CI = 1.94–13.71, *p* = 0.001), presence of B symptoms (OR = 2.56, 95%CI = 1.45–4.52, *p* = 0.001), and ECOG PS ≥2 (OR = 7.41, 95%CI = 1.49–36.92, *p* = 0.015) in PTCL.

**Conclusion:**

According to the present meta-analysis, higher sPD-L1 levels were significantly correlated with poor OS and inferior PFS in patients with PTCL. Additionally, high sPD-L1 levels were also associated with clinical features representing the development of PTCL.

## KEY MESSAGES

1. The present meta-analysis has first explored the impact of sPD-L1 on forecasting PTCL prognosis.
2. Higher sPD-L1 level was significantly correlated with dismal OS and inferior PFS of PTCL patients.
3. High sPD-L1 was also connected to clinical features representing the disease development of PTCL.

## Introduction

Peripheral T-cell lymphoma (PTCL) is a non-Hodgkin lymphoma characterized by diverse biological features and aggressive behavior [1]. PTCLs are rare and heterogeneous, representing 10% to 15% of all NHL cases, and encompass 28 distinct entities with distinct normal counterparts [2]. According to the latest classification of lymphoid neoplasms, PTCL comprises various subtypes with complex clinicopathologic features [3]. One of the most common PTCL subtypes is PTCL, not otherwise specified (PTCL-NOS), anaplastic large cell lymphoma, angioimmunoblastic T-cell lymphoma, and extranodal natural killer T-cell lymphoma (ENKTL)[4]. The clinical course of most PTCLs is aggressive and has a poor prognostic outcome. PTCL is managed through combination chemotherapy, typically CHOP (cyclophosphamide, doxorubicin, vincristine, and prednisone), CHOP-like regimen, or CHOEP (cyclophosphamide, doxorubicin, vincristine, etoposide, and prednisone) [5]. However, some patients experience disease progression during or immediately after treatment [6]. Despite combination chemotherapy, nodal PTCLs have dismal 5-year overall survival (OS) and progression-free survival (PFS) rates of approximately 35% and 25%, respectively [5]. Consequently, it is important to identify effective prognostic biomarkers for patients with PTCL.

As membrane-bound co-inhibitory immune checkpoint receptors, programmed death protein-1 (PD-1) and programmed death protein ligand-1 (PD-L1) are distributed in different human tumors and immune cells [7]. The PD-1/PD-L1 mechanism suppresses cytotoxic T cell activity, leading to programmed cell death and functional fatigue, thereby aiding tumor cells in evading the immune response [8]. Recently, soluble PD-L1 (sPD-L1) within the plasma or serum has been identified as a prognostic marker in different cancer types, such as prostate cancer [9], melanoma [10], renal cell carcinoma [11], glioma [12], and non-small cell lung cancer (NSCLC) [13]. The significance of sPD-L1 in forecasting PTCL prognosis has been widely explored; however, the results remain controversial [14–20]. Some studies have reported that elevated sPD-L1 significantly predicts PTCL prognosis [14, 15, 19, 20]. Nonetheless, in some studies, sPD-L1 was not related to survival in PTCL [18]. Therefore, we conducted this meta-analysis to examine the precise significance of sPD-L1 in predicting PTCL prognosis. Additionally, the association between sPD-L1 and clinicopathological characteristics of PTCL was explored.

## Materials and methods

### Study guideline

We performed this study in line with the preferred reporting items for systematic reviews and meta-analyses (PRISMA) guidelines [21].

### Search strategy

We searched PubMed, Embase, Web of Science, and Cochrane Library databases until July 31, 2024, using the following search strategies: (serum, plasma, blood, blood serum, soluble or circulating), (PD-L1 or programmed cell death-ligand 1 or B7-H1 or B7 homolog 1 or CD274 or cluster of differentiation 274), and lymphoma. Free words and Medical Subject Headings terms were used in this study. The language of this study was restricted to English. In addition, we manually searched the references of pertinent studies to identify additional related studies.

### Eligibility criteria

The following studies were included: (1) plasma and serum levels of sPD-L1 were detected using enzyme-linked immunosorbent assay (ELISA); (2) patients were pathologically diagnosed with PTCL; (3) patients do not have any other immune systemic comorbidities such as inflammation, infection, or autoimmune diseases; (4) studies reporting the relationship between sPD-L1 and PTCL survival; (5) hazard ratios (HRs) and 95% confidence intervals (CIs) were computable or available; (6) a threshold for sPD-L1 levels was available; and (7) English studies. The following studies were excluded: (1) meeting abstracts, comments, reviews, case reports, and letters; (2) studies including duplicate patients; and (3) animal studies.

### Data acquisition and quality evaluation

Two reviewers (YW and YZ) independently screened the literature and extracted data from the eligible articles. Any discrepancy was settled through negotiation until a consensus was reached. The following data were collected: first author, country, year, sample size, sex, study design, study center, study period, histology, Ann Arbor stage, treatment, cut-off values, determination methods for threshold, specimen, survival endpoints, follow-up, survival analysis, HRs, and 95%CIs. The Newcastle–Ottawa scale (NOS) was used to assess study quality[22]. NOS scores were 0–9 points, with NOS ≥6 representing high quality.

### Statistical analysis

This study determined the pooled HRs and 95%CIs in predicting the value of sPD-L1 for predicting PTCL prognosis. *I*^2^ and chi-square-based Q statistics were used to assess heterogeneity across studies. An *I*^2^ > 50% or *p* < 0.10 indicates significant heterogeneity; therefore, a random-effects model was applied; otherwise, a fixed-effects model was selected. A subgroup analysis was performed to explore the prognostic role of sPD-L1 in various patient populations. Additionally, the relationships between sPD-L1 and PTCL clinicopathological characteristics were evaluated by combining the odds ratios (ORs) with 95%CIs. By sequentially excluding individual datasets, we performed sensitivity analysis to ensure stable and creditable meta-analysis findings. Egger’s and Begg’s tests were used to examine the potential publication bias. Statistical analysis was performed using Stata 12.0 (Stata Corp LP, College Station, TX, USA). Statistical significance was set at *p* < 0.05.

## Results

### Literature retrieval procedure

**Figure 1.**
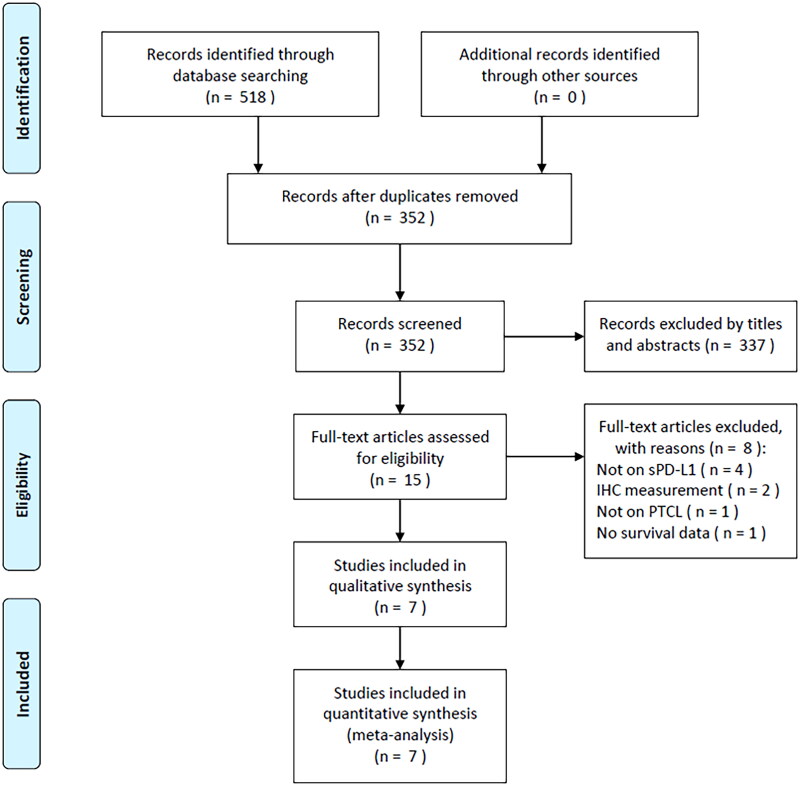
The PRISMA flow diagram of identifying eligible studies.

The initial literature search identified 518 articles, of which 352 were retained after duplicate removal (Figure 1). Through title and abstract review, there were 337 articles eliminated due to irrelevance. As a result, 15 articles were examined by reading the full texts. Subsequently, there were eight articles excluded because of irrelevance to sPD-L1 (*n* = 4), PD-L1 detected by immunohistochemistry (*n* = 2), not on PTCL (*n* = 1), and no survival data provided (*n* = 1). Ultimately, seven studies with 445 patients [14–20] were recruited for this study (Figure 1).

### Study features

Table 1 displays the basic study features [14–20] in this meta-analysis. All studies had a single-center design and were published between 2016 and 2022. There were five articles conducted in China [14, 15, 17–19] and one each conducted in Japan [16] and Korea [20], respectively. Six articles had a retrospective design [14, 16–20], while one had a prospective design [15], with a sample size of 17–107 (median, 77). Three articles recruited ENKTL patients [15, 19, 20]; two included PTCL-NOS patients [17, 18]; and one each enrolled natural killer/T-cell lymphoma (NKTL) [14] and nasal natural killer/T-cell lymphoma (NNKTL) cases [16]. Five studies recruited Ann Arbor stages I–IV cases [16–20] and two studies included stage I–II cases [14, 15]. Four studies treated patients with concurrent chemoradiotherapy (CCRT) [14–16, 19], two studies used chemotherapy [17, 18], and one study used immunotherapy [20]. All included studies used enzyme-linked immunosorbent assay (ELISA) to detect sPD-L1 levels and the detailed information are shown in Supplementary Table S1. The threshold sPD-L1 was 0.1–3.4 ng/ml (median value, 0.85 ng/ml). Four studies examined the threshold using the receiver-operating characteristic (ROC) curve [14–17], two studies applied the median value [18, 20], and one used X-tile software [19]. All seven studies reported the relationship between sPD-L1 and OS [14–20], while five showed the value of sPD-L1 in predicting PFS [14, 15, 17–19] in PTCL. Five articles obtained the HRs and 95%CIs through univariate regression [15, 16, 18–20] and two studies used multivariate regression [14, 17]. NOS scores were 7–9, suggesting that our enrolled articles were of high quality.

**Table 1.** Basic characteristics of included studies in this meta-analysis.

Study	Year	Country	Sample size	Gender (M/F)	Age (years) Median (range)	Study design	Study period	Disease type	Ann Arbor stage	Treatment	Cut-off value (ng/ml)	Cut-off determination	Method of detection	Specimen	Survival endpoints	PFS survival rate (%) sPD-L1 high vs sPD-L1 low	OS survival rate (%) sPD-L1 high vs sPD-L1 low	Follow-up (months) Median(range)	Survival analysis	NOS score
Bi, X. W.	2016	China	77	42/35	≤60 y: 66 >60 y: 11	Retrospective	2008–2015	NKTL	I-II	CCRT	3.4	ROC curve	ELISA	Serum	OS, PFS	5-year: 24.2% vs 81.3%	5-year: 36.5% vs 91.7%	38.0(9.4–79.0)	Multivariate	7
Wang, H.	2016	China	97	53/44	42	Prospective	2008–2015	ENKTL	I-II	CCRT	3.23	ROC curve	ELISA	Serum	OS, PFS	5-year: 19.5% vs 86.7%	5-year: 44.6% vs 92.5%	1–80	Univariate	9
Nagato, T.	2017	Japan	17	13/4	58(20–85)	Retrospective	2000–2014	NNKTL	I-IV	CCRT	0.85	ROC curve	ELISA	Serum	OS	NR	5-year: 57.4% vs 95.6%	1–60	Univariate	7
Shen, H.	2019	China	80	46/34	46.5(18–78)	Retrospective	2016–2018	PTCL-NOS	I-IV	Chemotherapy	0.176	ROC curve	ELISA	Plasma	OS, PFS	20-month: 0 vs 55.7%	20-month: 37.6% vs 90.5%	10(2–20)	Multivariate	8
Zhang, X.	2019	China	37	20/17	56(16–77)	Retrospective	2013–2016	PTCL-NOS	I-IV	Chemotherapy	1.696	Median value	ELISA	Plasma	OS, PFS	5-year: 25.6% vs 83.2%	5-year: 42.1% vs 91.4%	71	Univariate	7
Li, J. W.	2020	China	107	84/23	44(17–76)	Retrospective	2010–2017	ENKTL	I-IV	CCRT	0.219	X-tile	ELISA	Plasma	OS, PFS	5-year: 36.6% vs 72.9%	5-year: 57.3% vs 92.8%	65(2–119)	Univariate	8
Kim, S. J.	2022	Korea	30	21/9	51(23–75)	Retrospective	2017–2021	ENKTL	I-IV	Immunotherapy	0.1	Median value	ELISA	Plasma	OS	NR	24-month: 7.5% vs 42.6%	1–30	Univariate	8

NKTL: natural killer/T-cell lymphoma; ENKTL: extranodal natural killer/T-cell lymphoma; NNKTL: nasal natural killer/T-cell lymphoma; PTCL: peripheral T-cell lymphoma; PTCL-NOS: PTCL-not otherwise specified; CCRT: concurrent chemoradiotherapy; ROC: receiver-operating characteristic; OS: overall survival; PFS: progression-free survival; NOS: Newcastle-Ottawa Scale; NR, not reported.

### sPD-L1 and OS

**Figure 2.**
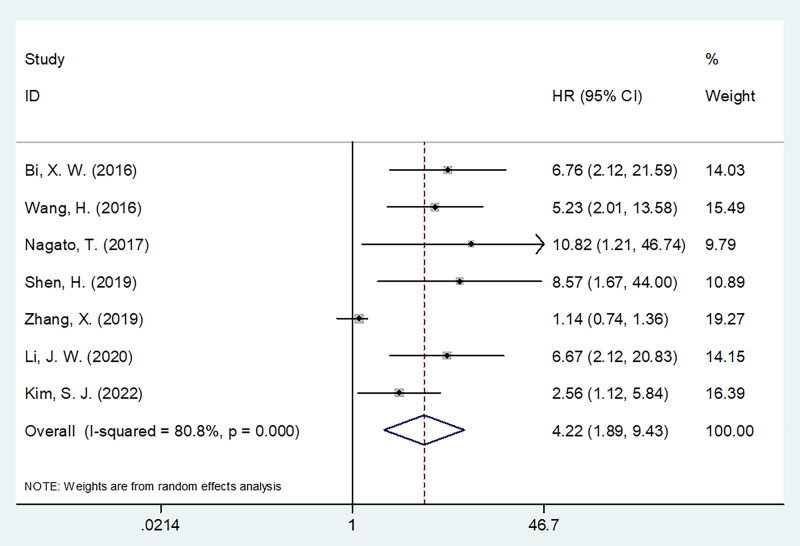
Forest plots showing the pooled HR with 95% CI of prognostic value of sPD-L1 for OS in PTCL.

Seven studies with 445 patients [14–20] provided the data on relationship between sPD-L1 and OS in PTCL. A random-effects model was used because of obvious heterogeneity (I2 = 80.8%, *p* < 0.001). The combined findings were HR = 4.22, 95%CI = 1.89–9.43, *p* < 0.001, suggesting an obvious association between elevated sPD-L1 and OS in PTCL (Table 2, Figure 2). Based on subgroup analyses, the significant role of sPD-L1 in predicting OS remained unaffected by sample size, country, study design, clinical stage, specimen, and survival analysis type (*p* < 0.05, Table 2). Moreover, subgroup analysis also demonstrated that sPD-L1 still markedly predicted dismal OS in the ENKTL (*p* < 0.001), NKTL (*p* = 0.001), NNLTL (*p* = 0.011), CCRT treatment (*p* < 0.001), immunotherapy (*p* = 0.026), cut-off value <1.0 ng/ml (*p* < 0.001), cut-off analysis based on ROC curve (*p* < 0.001), and X-tile (*p* = 0.001) subgroups (Table 2).

**Table 2.** Subgroup analysis of prognostic value of sPD-L1 for OS in patients with PTCL.

Subgroups	No. of studies	No. of patients	Effects model	HR (95%CI)	*p*	Heterogeneity	
						*I*^2^(%)	Ph
Total	7	445	Random	4.22(1.89–9.43)	<0.001	80.8	<0.001
Country							
China	5	398	Random	4.27(1.49–12.23)	0.007	84.9	<0.001
Japan	1	17	–	10.82(1.74–67.25)	0.011	–	–
Korea	1	30	–	2.56(1.12–5.84)	0.026	–	–
Sample size							
<80	4	161	Random	3.09(1.14–8.36)	0.026	80.7	0.001
≥80	3	284	Fixed	6.17(3.16–12.04)	<0.001	0	0.865
Study design							
Prospective	1	97	–	5.23(2.01–13.58)	0.001	–	–
Retrospective	6	348	Random	4.08(1.67–9.95)	0.002	80.8	<0.001
Disease type							
ENKTL	3	234	Fixed	4.04(2.33–6.98)	<0.001	8.8	0.334
PTCL-NOS	2	117	Random	2.65(0.38–18.56)	0.326	82.2	0.018
NKTL	1	77	–	6.76(2.12–21.57)	0.001	–	–
NNKTL	1	17	–	10.82(1.74–67.25)	0.011	–	–
Ann Arbor stage							
I-II	2	174	Fixed	5.80(2.77–12.12)	<0.001	0	0.737
I-IV	5	271	Random	3.66(1.41–9.49)	0.008	80.0	<0.001
Treatment							
CCRT	4	298	Fixed	6.42(3.57–11.54)	<0.001	0	0.918
Chemotherapy	2	117	Random	2.65(0.38–18.56)	0.328	82.2	0.018
Immunotherapy	1	30	–	2.56(1.12–5.84)	0.026	–	–
Cut-off value (ng/ml)							
<1.0	4	234	Fixed	4.46(2.48–8.03)	<0.001	19.6	0.292
≥1.0	3	211	Random	3.16(0.87–11.50)	0.081	87.5	<0.001
Cut-off determination							
ROC curve	4	271	Fixed	6.62(3.52–12.44)	<0.001	0	0.894
Median value	2	67	Random	1.55(0.72–3.34)	0.259	68.8	0.073
X-tile	1	107	–	6.67(2.13–20.90)	0.001	–	–
Specimen							
Serum	3	191	Fixed	6.33(3.19–12.54)	<0.001	0	0.780
Plasma	4	254	Random	3.02(1.14–7.97)	0.026	80.7	0.001
Survival analysis							
Univariate	5	288	Random	3.45(1.41–8.44)	0.007	81.8	<0.001
Multivariate	2	157	Fixed	7.32(2.84–18.86)	<0.001	0	0.816

NKTL: natural killer/T-cell lymphoma; ENKTL: extranodal natural killer/T-cell lymphoma; NNKTL: nasal natural killer/T-cell lymphoma; PTCL: peripheral T-cell lymphoma; PTCL-NOS: PTCL-not otherwise specified; CCRT: concurrent chemoradiotherapy; OS: overall survival.

### sPD-L1 and PFS

**Figure 3.**
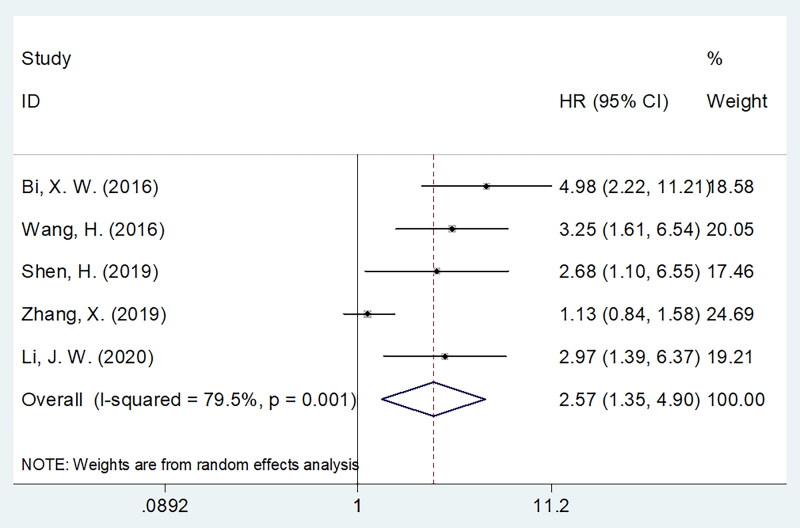
Forest plots showing the pooled HR with 95% CI of prognostic value of sPD-L1 for PFS in PTCL.

Five studies comprising 398 patients [14, 15, 17–19] reported the significance of sPD-L1 in predicting PFS. Based on our combined findings, higher sPD-L1 levels were significantly correlated with poor PFS in PTCL (HR = 2.57, 95%CI = 1.35–4.90, *p* = 0.004; Table 3, Figure 3). Subgroup analysis revealed that sPD-L1 significantly predicted poor PFS, irrespective of the study design (Table 3). Furthermore, sPD-L1 was still the factor for predicting worse PFS of sample size ≥80, ENKTL, NKTL, stage I-II, CCRT treatment, cut-off value <1.0 ng/ml, cut-off determination of ROC curve and X-tile, specimen of serum, and multivariate survival analysis subgroups (all *p* < 0.05; Table 3).

**Table 3.** Subgroup analysis of prognostic value of sPD-L1 for PFS in patients with PTCL.

Subgroups	No. of studies	No. of patients	Effects model	HR (95%CI)	*P*	Heterogeneity	
						I^2^(%)	Ph
Total	5	398	Random	2.57(1.35–4.90)	0.004	79.5	0.001
Sample size							
<80	2	114	Random	2.25(0.53–9.66)	0.273	91.1	0.001
≥80	3	284	Fixed	3.00(1.92–4.69)	<0.001	0	0.946
Study design							
Prospective	1	97	–	3.25(1.61–6.54)	0.001	–	–
Retrospective	4	301	Random	2.44(1.14–5.25)	0.022	81.4	0.001
Disease type							
ENKTL	2	204	Fixed	3.11(1.86–5.22)	<0.001	0	0.865
PTCL-NOS	2	117	Random	1.56(0.69–3.57)	0.288	68.9	0.073
NKTL	1	77	–	4.98(2.22–11.19)	<0.001	–	–
Ann Arbor stage							
I-II	2	174	Fixed	3.90(2.29–6.62)	<0.001	0	0.433
I-IV	3	224	Random	1.91(0.93–3.95)	0.079	73.6	0.023
Treatment							
CCRT	3	281	Fixed	3.57(2.31–5.51)	<0.001	0	0.623
Chemotherapy	2	117	Random	1.56(0.69–3.57)	0.288	68.9	0.073
Cut-off value (ng/ml)							
<1.0	2	187	Fixed	2.84(1.59–5.08)	<0.001	0	0.865
≥1.0	3	211	Random	2.50(0.94–6.65)	0.067	87.6	<0.001
Cut-off determination							
ROC curve	3	254	Fixed	3.54(2.24–5.58)	<0.001	0	0.573
Median value	1	37	–	1.13(0.82–1.54)	0.467	–	–
X-tile	1	107	–	2.97(1.38–6.36)	0.005	–	–
Specimen							
Serum	2	174	Fixed	3.90(2.29–6.62)	<0.001	0	0.433
Plasma	3	224	Random	1.91(0.93–3.95)	0.079	73.6	0.023
Survival analysis							
Univariate	3	241	Random	2.09(0.95–4.60)	0.066	81.6	0.004
Multivariate	2	157	Fixed	3.77(2.07–6.86)	<0.001	1.5	0.314

NKTL: natural killer/T-cell lymphoma; ENKTL: extranodal natural killer/T-cell lymphoma; PTCL: peripheral T-cell lymphoma; PTCL-NOS: PTCL-not otherwise specified; CCRT: concurrent chemoradiotherapy; ROC: receiver-operating characteristic; PFS: progression-free survival.

### Association of sPD-L1 with clinicopathological features

**Figure 4.**
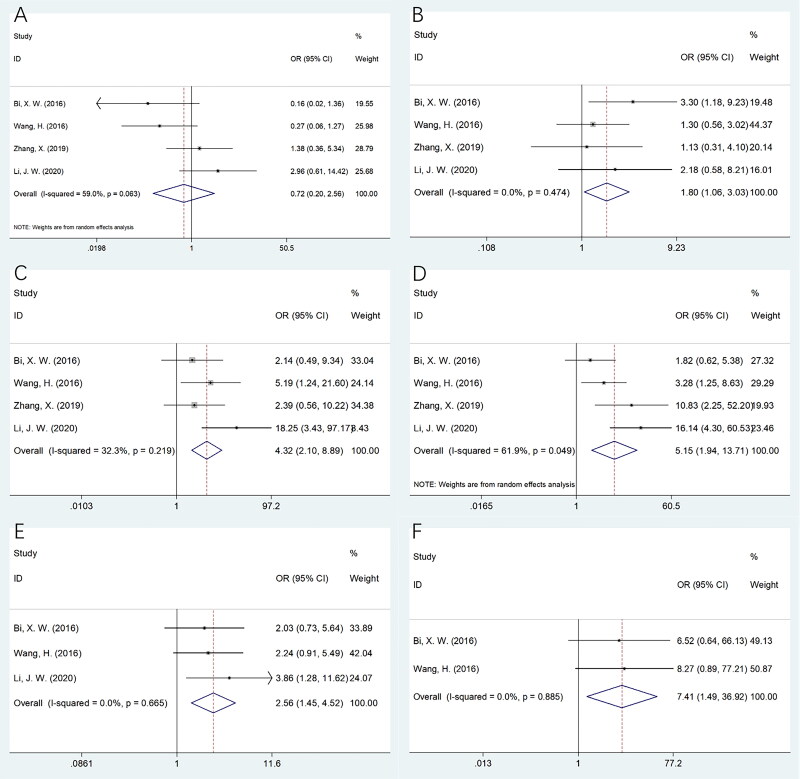
The association between sPD-L1 and clinicopathological features of PTCL. (A) Age (years) (≥60 vs <60); (B) Gender (male vs female); (C) IPI score (≥2 vs 0–1); (D) LDH level (elevated vs normal); (E) B symptoms (yes vs no); and (F) ECOG PS (≥2 vs 0–1).

Four studies, including 318 patients [14, 15, 18, 19] mentioned the association of sPD-L1 with PTCL clinicopathological features. As revealed by the pooled data, higher sPD-L1 levels were markedly correlated with male sex (OR = 1.80, 95%CI = 1.06–3.03, *p* = 0.029), International Prognostic Index (IPI) score ≥2 (OR = 4.32, 95%CI = 2.10–8.89, *p* < 0.001), elevated lactate dehydrogenase (LDH) level (OR = 5.15, 95%CI = 1.94–13.71, *p* = 0.001), presence of B symptoms (OR = 2.56, 95%CI = 1.45–4.52, *p* = 0.001), and ECOG PS ≥2 (OR = 7.41, 95%CI = 1.49–36.92, *p* = 0.015) (Table 4; Figure 4). However, sPD-L1 was not closely related to PTCL age (OR = 0.72, 95%CI = 0.20–2.56, *p* = 0.613; Table 4; Figure 4).

**Table 4.** The correlation between sPD-L1 and clinicopathological features in patients with PTCL.

Variables	No. of studies	No. of patients	Effects model	OR (95%CI)	*p*	Heterogeneity	
						*I*^2^(%)	Ph
Age (years) (≥60 vs <60)	4	318	Random	0.72(0.20–2.56)	0.613	59.0	0.063
Gender (male vs female)	4	318	Fixed	1.80(1.06–3.03)	0.029	0	0.474
IPI score (≥2 vs 0–1)	4	318	Fixed	4.32(2.10–8.89)	<0.001	32.3	0.219
LDH level (elevated vs normal)	4	318	Random	5.15(1.94–13.71)	0.001	61.9	0.049
B symptoms (yes vs no)	3	281	Fixed	2.56(1.45–4.52)	0.001	0	0.665
ECOG PS (≥2 vs 0–1)	2	174	Fixed	7.41(1.49–36.92)	0.015	0	0.885

PTCL: peripheral T-cell lymphoma; IPI: International Prognostic Index; LDH: lactate dehydrogenase; ECOG PS: Eastern Cooperative Oncology Group performance status.

### Sensitivity analysis

**Figure 5.**
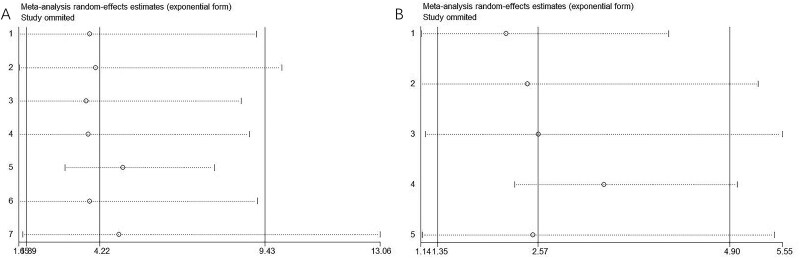
Sensitivity analysis. (A) OS and (B) PFS.

A sensitivity analysis was performed to determine the influence of individual studies on the overall analysis. The combined meta-analysis effect of sPD-L1 on OS and PFS was not substantially changed by excluding any of the included studies, which indicated the robustness of our results (Figure 5).

### Publication bias

**Figure 6.**
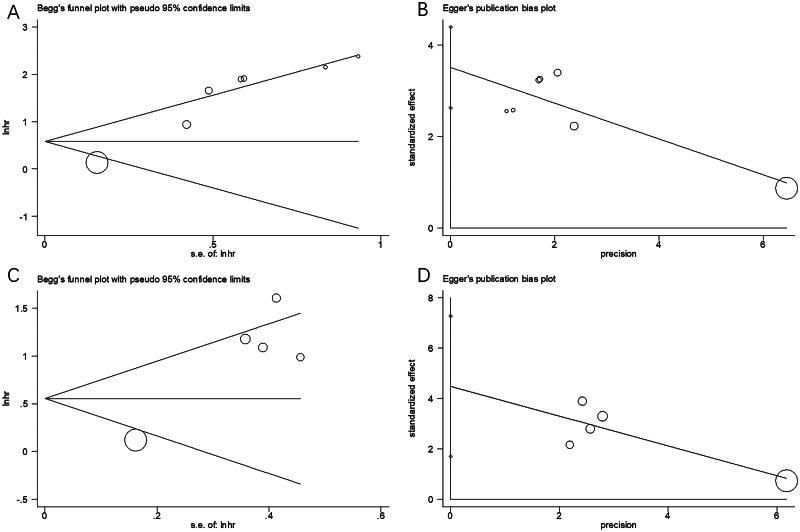
Publication bias test. (A) Begg’s test for OS, *p* = 0.368; (B) Egger’s test for OS, *p* = 0.105; (C) Begg’s test for PFS, *p* = 0.806; and (D) Egger’s test for PFS, *p* = 0.095.

We conducted Begg’s and Egger’s tests to analyze possible publications. The results indicated no significant publication bias for OS (Begg’s test, *p* = 0.368; Egger’s test, *p* = 0.105) or PFS (Begg’s test, *p* = 0.806; Egger’s test, *p* = 0.095) (Figure 6).

### Discussion

The effect of sPD-L1 on PTCL prognosis has been widely examined, but inconsistent results have been reported. In our study, we aggregated data from seven articles involving 445 cases. Our results indicated that elevated sPD-L1 levels significantly predicted dismal OS and PFS in PTCL. Moreover, in this meta-analysis, higher sPD-L1 level was significantly associated with male sex, IPI score ≥2, high LDH level, B symptoms, and ECOG PS ≥2 in PTCL. As verified by the publication bias and sensitivity analyses, the findings were stable. Collectively, in the present study, sPD-L1 was a significant and reliable blood-based biomarker of survival outcomes in PTCL. The present meta-analysis is the first to explore the impact of sPD-L1 on the prognosis of PTCL.

Although the precise mechanisms underlying the effect of sPD-L1 on PTCL prognosis are largely unclear, they are interpreted as follows. First, there is no clear understanding of the origin of circulating sPD-L1 levels. The sPD-L1 level was recently found to be unrelated to tissue PD-L1 expression, indicating that tissue expression may not be the main sPD-L1 source [23, 24]. It is known that tumor cells produce PD-L1; therefore, serum sPD-L1 derived from tumor cells is likely to be substantial in patients with high tumor burdens [25]. Second, other sources of sPD-L1 besides proteolytic products include high levels of expression of various sPD-L1 isoforms due to sPD-L1 alternative splicing variants, serum expression exomes, and dendritic cell secretion [26, 27]. Exosomes in the supernatant of cultured cells are involved in the release of PD-L1 and have been shown to be induced by IFN-γ addition to the media [28]. Third, PD-1/PD-L1 activation causes a series of effects in the T-cell response, such as an enhanced threshold of activated T cells, reduced proliferation, and apoptosis of exhausted T cells [29]. Furthermore, sPD-L1 can suppress immunity both locally and globally in the tumor microenvironment and peripheral blood [30]. By targeting secondary lymphoid organs, sPD-L1 regulates systemic antitumor immune responses by inhibiting IFN-γ secretion by T cells [31].

Notably, in this meta-analysis, three studies detected the sPD-L1 level in serum [14–16], and four studies used plasma [17–20]. Serum is the liquid that remains after the blood has clotted. Plasma is the liquid that remains when clotting is prevented with the addition of an anticoagulant. There are statistical solutions to reducing serum plasma differences [32]. In this meta-analysis, we performed a subgroup analysis based on the specimen, and the prognostic value of sPD-L1 for OS was not influenced by the specimen (Table 2).

There could be significant variations in the immune status of PTCL patients, with sPD-L1 levels potentially indicating this status [33, 34]. The sPD-L1 levels could also change in other diseases such as coronary artery disease [33], myelodysplastic syndromes [34], and COVID-19 infection [35]. We excluded those patients with immune-related diseases in eligibility criteria in this meta-analysis.

According to the World Health Organization (WHO), PTCL is grouped into nearly 30 types [36]. PTCL is a heterogenous disease with various molecular and histopathological classifications [37]. In this meta-analysis, we included patients with various subtypes of PTCL including ENKTL, PTCL-NOS, NKTL, and NNKTL cases (Table 1). Most included patients were diagnosed with NKTL (328 out of 445), and we, therefore, conducted subgroup analysis based on disease type (Tables 2 and 3). The results showed that sPD-L1 was a significant prognostic marker for OS and PFS in patients with NKTL, but not in PTCL-NOS (Tables 2 and 3). This subgroup analysis quantitatively revealed the prognostic efficiency of sPD-L1 in distinct subtypes of PTCL.

Recently, the value of sPD-L1 for predicting cancer prognosis has been widely explored by meta-analysis [38–41]. Zhang et al. conducted a meta-analysis involving 26 studies and found that sPD-L1 levels were markedly associated with poor PFS and OS in NSCLC patients receiving immune checkpoint inhibitors (ICIs) [38]. Xue et al. reported that higher sPD-L1 levels were related to poor OS and disease-free survival/recurrence-free survival/tumor-free survival/time to progression in hepatocellular carcinoma in a meta-analysis of 1291 patients [39]. According to a recent meta-analysis, a higher sPD-L1 level is related to poor OS and PFS in NSCLC [40]. As reported by Széles et al., the poor OS of cancer patients receiving ICI therapy was closely related to the higher sPD-L1 level before treatment in a meta-analysis including 1054 cases [41]. Our results conformed with the sPD-L1 results for other cancer types.

This study had certain limitations. First, the sample size is relatively small. Only 445 patients were enrolled in the present study, although we retrieved the most recent literature. This may be due to the rarity of PTCL, with an annual prevalence of 0.4–0.5/100,000 individuals in the USA [42]. Second, all included patients were from Asian countries and non-Asian patients were not included. The study region was not restricted, and all the included studies were published in English. Third, the threshold sPD-L1 level was not uniform among the enrolled articles, probably causing selection bias. Fourth, PD-1 was not reported in included studies. PD-L1 and PD-1 can both be expressed in PTCL. It would be helpful if some studies investigated the association between the levels of these proteins and their soluble forms. Therefore, owing to these limitations, large multicenter studies should be conducted to validate these results.

## Conclusions

In summary, this meta-analysis demonstrated that elevated sPD-L1 levels were significantly related to poor OS and inferior PFS in PTCL patients. In addition, high sPD-L1 levels were also associated with clinical features representing the development of PTCL.

## Supplementary Material

Supplementary Table S1.docx

PRISMA_2020_checklist.docx

## Data Availability

The data that support the findings of this study are available from the corresponding author upon reasonable request.
