# Supplementary material for: Prognostic and clinicopathological value of soluble programmed cell death ligand-1 (sPD-L1) in patients with peripheral T-cell lymphoma: a meta-analysis
Source: Ann Med. 2025 Feb 10;57(1):2458236. doi: 10.1080/07853890.2025.2458236 (PMC11812115; doi:10.1080/07853890.2025.2458236)
Supplement: Supplementary Table S1.docx [file IANN_A_2458236_SM1228.docx]

Supplementary Table S1 The detailed information on ELISA method to detect sPD-L1 level in included studies.

| Study | Year | Method of detection | The indicator | ELISA kit | ELISA kit manufacturer | Minimum detectable level (ng/ml) |
| --- | --- | --- | --- | --- | --- | --- |
| Bi, X. W. | 2016 | ELISA | sPD-L1 | PDCD1LG1 ELISA kit | Cloud-Clone Corp., Wuhan, China | NR |
| Wang, H. | 2016 | ELISA | sPD-L1 | PDCD1LG1 ELISA kit | USCN Life Science, Wuhan, China | 0.057 |
| Nagato, T. | 2017 | ELISA | sPD-L1 | PDCD1LG1 ELISA Kit | USCN Life Science Inc. | 0.013 |
| Shen, H. | 2019 | ELISA | sPD-L1 | Abcam ab 214565 | Abcam | 0.0218 |
| Zhang, X. | 2019 | ELISA | sPD-L1 | Human PD‐L1 ELISA kit DB7H10 | R&D Systems | NR |
| Li, J. W. | 2020 | ELISA | sPD-L1 | PD-L1 ELISA kit # DB7H10 | R&D Systems | 0.015 |
| Kim, S. J. | 2022 | ELISA | sPD-L1 | PD-L1 ELISA kit # DB7H10 | R&D systems, Minneapolis, MN, USA | 0.015 |

ELISA, enzyme-linked immunosorbent assay; NR, not reported;
